# Combination therapy targeting ectopic ATP synthase and 26S proteasome induces ER stress in breast cancer cells

**DOI:** 10.1038/cddis.2014.504

**Published:** 2014-11-27

**Authors:** H-Y Chang, T-C Huang, N-N Chen, H-C Huang, H-F Juan

**Affiliations:** 1 Department of Life Science, National Taiwan University, Taipei, Taiwan; 2 PhD Program for Cancer Biology and Drug Discovery, College of Medical Science and Technology, Taipei Medical University, Taipei, Taiwan; 3 Institute of Molecular and Cellular Biology, National Taiwan University, Taipei, Taiwan; 4 Institute of Biomedical Informatics, Center for Systems and Synthetic Biology, National Yang-Ming University, Taipei, Taiwan; 5 Graduate Institute of Biomedical Electronics and Bioinformatics, National Taiwan University, Taipei, Taiwan

## Abstract

F_1_F_o_ ATP synthase is present in all organisms and is predominantly located on the inner membrane of mitochondria in eukaryotic cells. The present study demonstrated that ATP synthase and electron transport chain complexes were ectopically expressed on the surface of breast cancer cells and could serve as a potent anticancer target. We investigated the anticancer effects of the ATP synthase inhibitor citreoviridin on breast cancer cells through proteomic approaches and revealed that differentially expressed proteins in cell cycle regulation and in the unfolded protein response were functionally enriched. We showed that citreoviridin triggered PERK-mediated eIF2*α* phosphorylation, which in turn attenuated general protein synthesis and led to cell cycle arrest in the G_0_/G_1_ phase. We further showed that the combination of citreoviridin and the 26S proteasome inhibitor bortezomib could improve the anticancer activity by enhancing ER stress, by ameliorating citreoviridin-caused cyclin D_3_ compensation, and by contributing to CDK1 deactivation and PCNA downregulation. More interestingly, the combined treatment triggered lethality through unusual non-apoptotic caspase- and autophagy-independent cell death with a cytoplasmic vacuolization phenotype. The results imply that by boosting ER stress, the combination of ATP synthase inhibitor citreoviridin and 26S proteasome inhibitor bortezomib could potentially be an effective therapeutic strategy against breast cancer.

Breast cancer is the most common malignancy among women and is one of the leading causes of cancer deaths worldwide. More than 235 000 patients are diagnosed with breast cancer annually in the United States, and approximately 40 000 women are expected to die from the disease in 2014.^1, 2^ Treating breast cancer with a combination of treatment options, such as hormonal therapy, chemotherapy, radiation therapy, surgery, and targeted therapies aims to provide clinical benefits, to improve patients' quality of life, and to minimize side effects. However, an increase in the number of unresponsive and resistant cases for standard treatments, including aromatase inhibitors, estrogen receptor antagonists, human epidermal growth factor receptor 2-targeted monoclonal antibody, and taxane chemotherapies, has been reported.^3, 4, 5, 6, 7^ Therefore novel therapeutic biomarkers and new treatment options that overcome resistance are needed.

Adenosine triphosphate (ATP) synthase is a membrane-associated protein complex comprising two sectors: the water-soluble catalytic sector (F_1_) with the subunit composition *α*_3_*β*_3_*γδɛ*, and the membrane-bound proton-translocating sector (F_o_) with the subunit composition ab_2_c_10__–15._^8, 9^ ATP synthase catalyzes the phosphorylation of adenosine diphosphate to ATP through the proton-motive force generated by the electron transport chain (ETC) in energy-transducing membranes.^10, 11, 12^ ATP synthase is predominantly located on the membranes of mitochondria, bacteria, and chloroplast thylakoids. Recent studies have shown that ATP synthase is present on the plasma membrane (PM) of highly proliferating cell types in eukaryotes, including cancer cells,^13, 14^ endothelial cells,^15, 16^ keratinocytes,^17^ adipocytes,^18^ and hepatocytes.^19^

Previously, we demonstrated that an ATP synthase inhibitor causes cytotoxicity to breast cancer and lung cancer cells but not to normal cells,^13, 14^ suggesting that the ATP synthase inhibitor may be a potential drug for breast cancer therapy. To further understand the effects of the ATP synthase inhibitor on breast cancer cells, we performed proteomics approaches to investigate the differential expression profiles of breast cancer cells in response to ATP synthase inhibitor citreoviridin.

Citreoviridin is a toxic metabolite isolated from molds of the genera *Penicillium* and *Aspergillus*.^20^ Citreoviridin contains *α*-pyrone, a six-membered cyclic unsaturated ester that binds to the ATP synthase *β* subunit and causes toxicity to bacteria.^21, 22^ In the present study, we used citreoviridin to treat cancer cells and revealed the activation of the unfolded protein response (UPR) upon drug treatment.

The endoplasmic reticulum (ER) is responsible for protein folding, lipid and sterol biosynthesis, and intracellular calcium storage.^23^ Perturbations in ER homeostasis result in UPR by activating three ER-resident transmembrane transducers: inositol-requiring protein-1 (IRE1), protein kinase RNA (PKR)-like ER kinase (PERK), and activating transcription factor 6 (ATF6).^24, 25, 26, 27, 28^ Subsequently, phosphorylated PERK further phosphorylates Ser51 on the eukaryotic translation initiation factor 2*α* (eIF2*α*) and leads to general inhibition of translation and cell cycle arrest, thus preventing further influx of nascent proteins into the ER lumen.^29^ Simultaneously, dimerization and autophosphorylation of IRE1 and cleavage of ATF6 in the Golgi result in the gene expression of protein chaperones and components of the ER-associated degradation (ERAD) machinery.^30^

On the basis of cellular responses to ER stress, we intended to intensify cell death signaling by inducing the UPR and by inhibiting ERAD with citreoviridin and the proteasome inhibitor bortezomib, respectively. Consequently, we revealed the prompting of cytoplasmic vacuolization, which implies induction of a novel type of methuosis-like cell death.

## Results

### ATP synthase was ectopically expressed on the PM of mammary cancer cells

**Figure 1:**
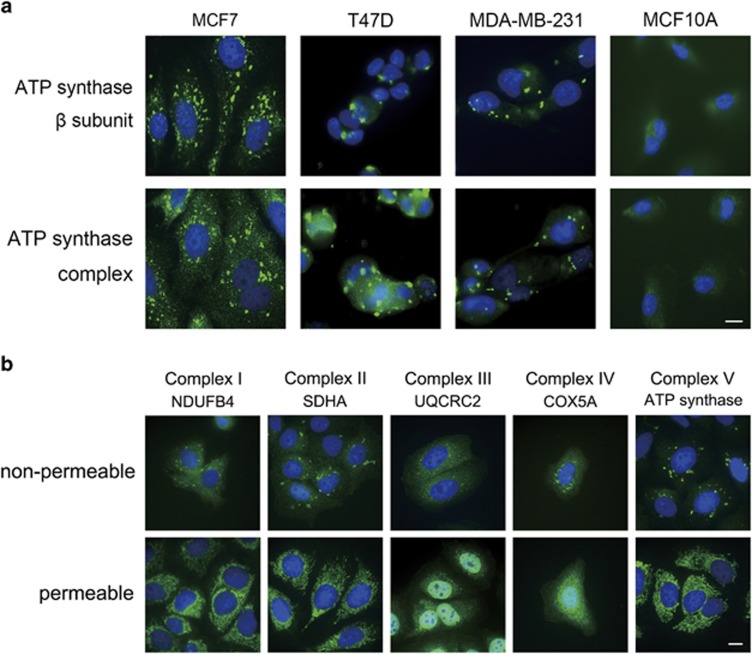
ATP synthase and ETC complexes were ectopically expressed on the PM of breast cancer cells. (**a**) 2 × 10^4^ breast cancer cells (MCF7, T47D, and MDA-MB-231) or non-tumorigenic breast cells (MCF10A) were seeded onto poly-L-lysine-coated coverslips. Cells were incubated with antibody-probing ATP synthase *β* subunit (upper) or with whole ATP synthase complex (lower). (**b**) MCF7 cells were incubated with antibody-probing ETC complex proteins NDUFB4, SDHA, UQRC2, COX5, or ATP synthase under permeable or nonpermeable conditions. Cells were subjected to labeling with anti-mouse IgG-Alexa488 (green) and then stained with nuclear DAPI (blue). Bars represent 10 *μ*m

To explore the ectopic expression of ATP synthase, we performed immunocytochemical tests on three breast cancer cell lines (i.e., MCF7, T47D, and MDA-MB-231) and non-oncogenic breast cells MCF10A. We observed punctate localization of ectopic ATP synthase on the PM of cancer cells but not on MCF10A cells (Figure 1a). Furthermore, the expression of ATP5B was observed in the PM fraction in MCF7, T47D, and MDA-MB-231 cells (Supplementary Figure S1). These results suggest that the specific expression of ectopic ATP synthase on cancer cells might be a potential drug-targeting signature for breast cancer.

### ETC complex proteins were ectopically expressed on the PM of MCF7 cells

To further investigate whether ETC was also ectopically expressed on the PM, we applied antibody-probing selected proteins from complexes I to IV to monitor the extracellular and mitochondrial distribution of the ETC and ATP synthase. We revealed the ectopic expression of complexes I–IV on the PM, but the complexes varied in pattern and size (Figure 1b). All of the ectopic expression in the non-permeable group was punctated, distinct from the network-like distribution in the permeable mitochondrial group, implying the ectopic ETC and ATP synthase might cluster at specific membrane structure of the PM.

### ATP synthase inhibitor citreoviridin inhibited proliferation of mammary cancer cells

**Figure 2:**
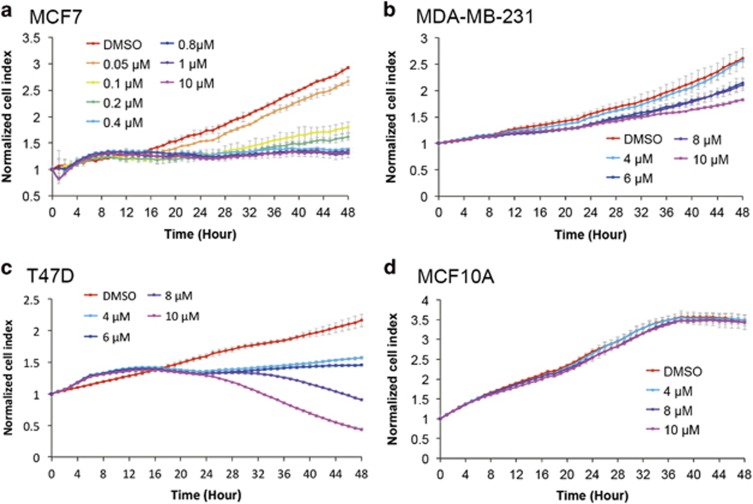
Citreoviridin inhibited cell proliferation of ectopic ATP synthase expressed on breast cancer cells. Real-time growth of (**a**) MCF7, (**b**) T47D, (**c**) MDA-MB-231, and (**d**) MCF10A cells after drug treatment was measured at hourly intervals for 48 h (*x* axis) by using the RTCA system. The normalized cell index (*y* axis) is presented as the mean±S.D.

To examine the inhibitory efficacy of citreoviridin against cell proliferation, we utilized a real-time cell analyzer to monitor cell responses to the drug over time. The results indicate that citreoviridin was cytotoxic to breast cancer cells MCF7, T47D, and MDA-MB-231 but not to the non-tumorigenic MCF10A (Figure 2). To evaluate the effect of citreoviridin on mitochondria, we examined the mitochondrial membrane potential (MMP) after 48-h treatment in MCF7 cells whose proliferation was inhibited by citreoviridin mostly. The result exhibited that the MMP was not affected during the course of the treatment (Supplementary Figure S2), implying the use of citreoviridin at the present dosage and treatment duration would at least not cause damages in mitochondrial function.

### Citreoviridin altered protein expression involving regulation of the cell cycle and ubiquitin-dependent protein modification

To determine the effects of citreoviridin on global protein expression, we utilized two-dimensional electrophoresis coupled with matrix-assisted laser desorption ionization time-of-flight tandem mass spectrometry. We found that 15 proteins were differentially expressed after 24 and 48 h of citreoviridin treatment, with protein pI values ranging from 4 to 7 and from 3 to 10 (Supplementary Figure S3 and Supplementary Table S1). Among the 15 identified proteins, 14 were significantly enriched in the protein–protein interaction (*P*=6.12 × 10^−6^) compared with 3.16 expected interactions within the genome background (Supplementary Figure S4). To analyze the major biological processes in which the identified proteins were involved, we applied gene ontology enrichment analysis. The analysis revealed that the most enriched biological processes were cell cycle phase transition (*P*=2.96 × 10^−6^), intracellular signaling (*P*=3.43 × 10^−6^), protein modification (*P*=6.57 × 10^−6^), regulation of apoptotic cell death (*P*=9.99 × 10^−6^), proteasome-mediated ubiquitin-dependent protein catabolism (*P*=1.52 × 10^−5^), response to unfolded proteins (*P*=1.65 × 10^−4^), and ER-associated ubiquitin-dependent protein catabolism (*P*=4.09 × 10^−4^).

### Citreoviridin arrested cell cycle progression in the G_0_/G_1_ phase

**Figure 3:**
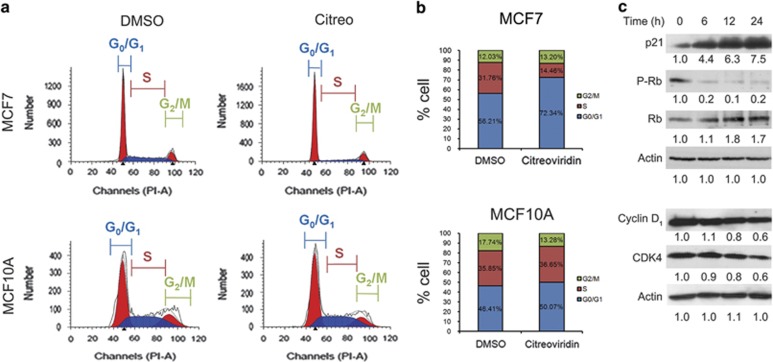
Citreoviridin caused cell cycle arrest in the G_o_/G_1_ phase. Cells were treated with 0.1 *μ*M citreoviridin for 48 h and then subjected to DNA content analysis. (**a**) DNA content was measured by using fluorescence-activated cell sorter, and the phase distribution was quantified with model fitting using ModFit. (**b**) Percentages were representative of cell cycle distributions from three replicate experiments in MCF7 (top) and MCF10A (bottom) cells. (**c**) MCF7 cells treated with 0.1 *μ*M citreoviridin were harvested at the indicated time points and analyzed by western blotting. Expression of each detected protein was normalized to the signal of the actin level at 0 h

Functional enrichment analysis of proteomic data indicated that citreoviridin might regulate cell cycle progression. To verify this effect, we analyzed the DNA content distribution in response to citreoviridin by flow cytometry. The results show that citreoviridin significantly led to an accumulation of DNA content in the G_0_/G_1_ phase from 56.21% to 72.34% after 48-h treatment in MCF7 but not in MCF10A (Figures 3a and b). Molecular evidence also indicates that citreoviridin augmented the expression of the cyclin-dependent kinase inhibitor (CKI) p21, reduced the protein expression of the G_0_/G_1_ cell cycle regulators cyclin-dependent kinase 4 (CDK4) and cyclin D_1_, and contributed to a decrease in retinoblastoma protein (Rb) phosphorylation in a time-dependent manner (Figure 3c), suggesting the inhibition of entry into the S phase in the cell cycle.

### Citreoviridin triggered UPR and PERK-mediated eIF2*α* phosphorylation

**Figure 4:**
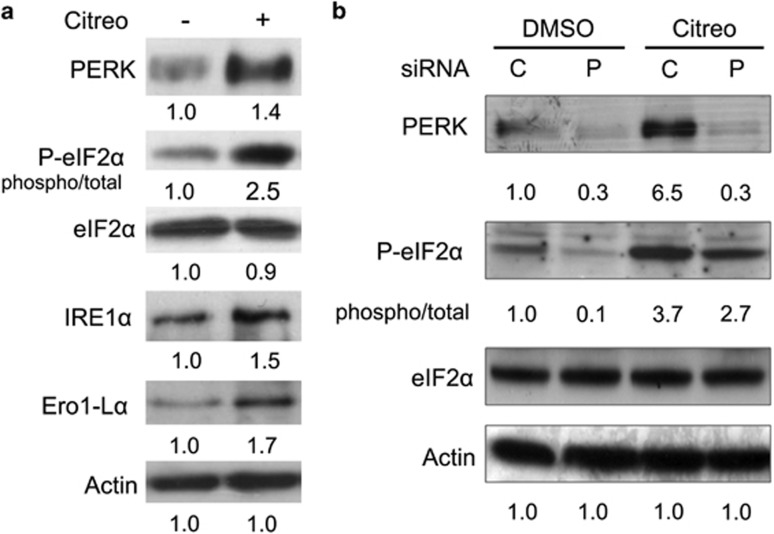
Citreoviridin triggered UPR- and PERK-mediated eIF2*α* phosphorylation. (**a**) Proteins from MCF7 cells treated with 0.1 *μ*M citreoviridin (+) or DMSO vehicle control (−) for 48 h were analyzed by western blotting. Expression of each detected protein was normalized to the signal of the actin level and the DMSO control. Phosphorylation of eIF2*α* was normalized to the total eIF2*α* level. (**b**) MCF7 cells were treated for 36 h with PERK (P) knockdown by siRNA or with a scrambled control (C) before treatment with 0.1 *μ*M citreoviridin (Citreo) or DMSO for 24 h. Expression of each detected protein was normalized to the signal of the actin level and of the cells transfected with the siRNA control and treated with DMSO

The functional enrichment analysis also emphasized the involvement of proteasome-mediated ubiquitin-dependent protein catabolism, response to unfolded proteins, and ER-associated ubiquitin-dependent protein catabolism. In response to the accumulation of misfolded proteins in the ER, cells activate the UPR to cope with the unfolded proteins. This occurs by inhibition of protein synthesis and by increasing chaperones and redox proteins to assist protein folding through a series of signaling from the ER lumen. We revealed that citreoviridin induced the UPR by triggering the protein expression or phosphorylation of PERK, eIF2*α*, IRE1*α*, and Ero1-L*α* (Figure 4a). We further demonstrated that small interfering RNA (siRNA) knockdown of PERK alleviated eIF2*α* phosphorylation (Figure 4b), implying that PERK activation mediates induction of eIF2*α* phosphorylation.

### Combination of citreoviridin and bortezomib suppressed cell proliferation and anchorage-dependent and -independent colony-forming ability

**Figure 5:**
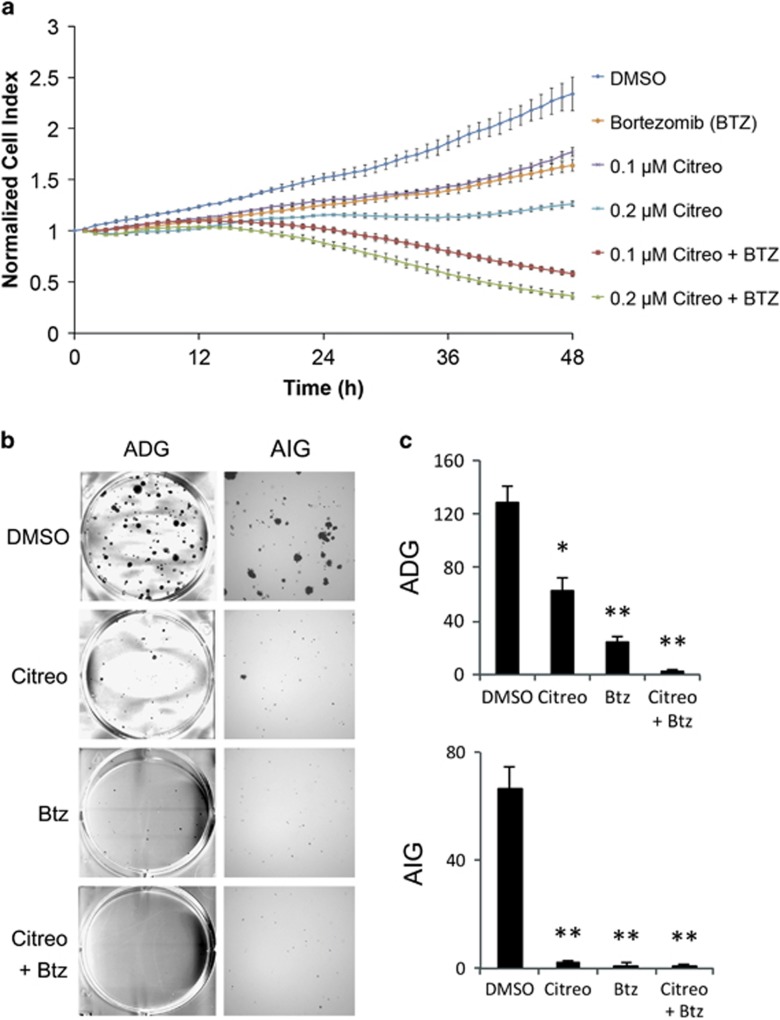
Combination of bortezomib and citreoviridin exhibited an additive effect on cytotoxicity. (**a**) Real-time cell growth of MCF7 cells was measured after drug treatment (*x* axis) at hourly intervals for 48 h by using an RTCA system and expressed as a normalized cell index (*y* axis). Cells were treated with 0.1 or 0.2 *μ*M citreoviridin (Citreo) in the presence or absence of 10 nM bortezomib (Btz). (**b**) Colony-formation assays were assessed through anchorage-dependent (left, ADG) or anchorage-independent (right, AIG) procedures, as described in the Materials and Methods section. Cells were treated with DMSO, 0.1 *μ*M citreoviridin (Citreo), 10 nM bortezomib (Btz), or a combination of 0.1 *μ*M Citreo and 10 nM Btz (Citreo+Btz). Colonies were grown in six-well plates and then stained with crystal violet. (**c**) The numbers of colonies of ADG in each well and AIG in each field per well were counted. Asterisks indicate significant differences between the control and treated group from three independent experiments (*P<0.01; **P<0.001)

To verify whether augmentation of ER stress by induction of UPR and inhibition of ERAD could intensify the cell death signal, we combined citreoviridin, which triggered the UPR we presented previously (Figure 4), and the proteasome-specific inhibitor bortezomib and measured their combined efficacy. The results show that citreoviridin and bortezomib exhibited an additive effect on MCF7 cell growth, with a combination index of 0.97 at 48-h co-treatment (Figure 5a). Additionally, we demonstrated that combination of citreoviridin and bortezomib reduced the colony-forming ability through anchorage-dependent and -independent routes (Figures 5b and c).

### Bortezomib decreased citreoviridin-caused cyclin D_3_ compensation and enhanced inhibition of entry into the S phase

**Figure 6:**
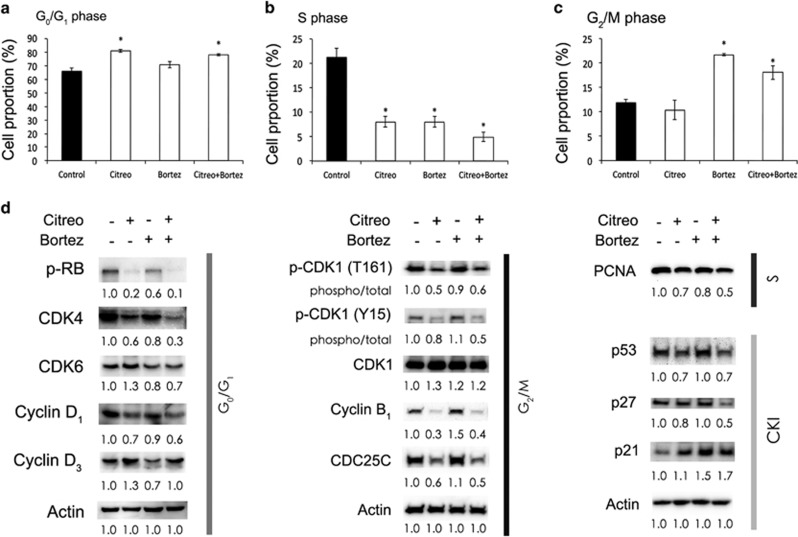
Combination of bortezomib and citreoviridin inhibited cell cycle progression through different cell cycle regulators. Cells treated with or without 0.1 *μ*M citreoviridin (Citreo) and 10 nM bortezomib (Btz) for 48 h were subjected to cell cycle analysis or harvested for western blotting analysis. Cell cycles distributed in the (**a**) G_0_/G_1_, (**b**) S, and (**c**) G_2_/M phases were quantified. *P-values of <0.01 show that treatment effects differed significantly from those on the DMSO control. (**d**) Protein expression levels of the cell cycle regulators and CKIs from cells treated for 48 h were normalized to actin and DMSO (−/−) levels. For the phosphorylated form of the indicated proteins, the level of phosphorylation was further normalized to the total level of the given protein

Subsequently, we analyzed the effects of bortezomib and citreoviridin on cell cycle progression and found that the combined treatment caused elevated populations in both the G_0_/G_1_ and G_2_/M phases accompanied by a significant reduction in the S phase (Figures 6a–c). The cell cycle regulators phospho-Rb, CDK4, cyclin D_1,_ phospho-CDK1, cyclin B_1_, Cdc25C, and proliferating cell nuclear antigen (PCNA) were inhibited by citreoviridin alone and in combination with bortezomib (Figure 6d). We also revealed that the combination of bortezomib and citreoviridin inhibited citreoviridin-induced compensation of cyclin D_3_ and CDK6, thus preventing Rb phosphorylation and S-phase entry by decreasing PCNA expression (Figure 6d). We moreover found that citreoviridin alone and in combination with bortezomib augmented only p21, whereas p53 and p27 were unaltered, showing that these two CKIs were not involved in citreoviridin- or bortezomib-induced cell cycle arrest (Figure 6d).

### The proteasome inhibitor bortezomib enhanced citreoviridin-induced UPR and cytoplasmic vacuolation

**Figure 7:**
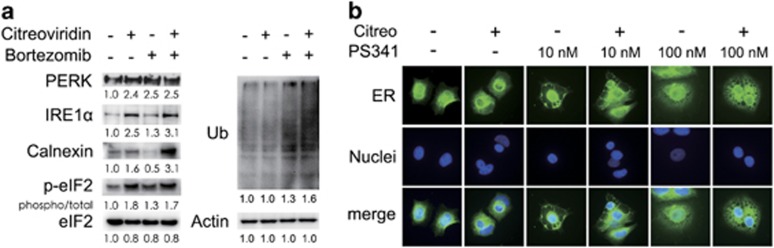
Combination of bortezomib and citreoviridin enhanced the UPR, protein ubiquitination, and cytoplasmic vacuolation. (**a**) Cells treated with or without 0.1 *μ*M citreoviridin (Citreo) and 10 nM bortezomib (Btz) for 48 h were harvested for western blotting analysis. Protein expression levels or total ubiquitinated protein (Ub) signals were normalized to actin and DMSO (−/−) levels, and the phosphorylated form of eIF2*α* was further normalized to the total eIF2*α* level. (**b**) Cells were seeded onto poly-L-lysine-coated coverslips and then treated with the indicated drugs for 5 days. ER and nuclei were labeled with calnexin (green) and DAPI (blue), respectively. Bar represents 10 *μ*m

Next, we demonstrated that the combination of citreoviridin and bortezomib enhanced the UPR by increasing PERK, IRE1*α*, and calnexin expression, as well as eIF2*α* phosphorylation and protein ubiquitination (Figure 7a). As the treatment period advanced to 5 days, we observed that prolonged UPR caused massive cytoplasmic vacuolation in the combined and bortezomib treatments (Figure 7b). The number of formed cytoplasmic vacuoles was higher in the combined treatment compared with that in the treatment with single agent or vehicle control. The results suggest that the combination of citreoviridin and bortezomib might induce non-apoptotic cell death, as highlighted by the cytoplasmic vacuolation.

### Both purinergic receptor signaling and UPR but not calcium influx are involved in citreoviridin-caused cell death

To investigate the influences of purinergic receptor signaling and UPR on citreoviridin-inhibited cell proliferation, ATP and chemical chaperone were applied after and prior to drug treatment, respectively. Citreoviridin-inhibited proliferation was partially recovered by ATP posttreatment and tauroursodeoxycholic acid pretreatment (Supplementary Figure S5). The involvement of extracellular ATP on P2Y ATP-gated ion channel was further investigated. We utilized calcium indicator to monitor the kinetics of calcium influx and found that the addition of citreoviridin or bortezomib did not change the intracellular calcium (Supplementary Figure S6a) and affect the ATP-boosted calcium influx (Supplementary Figure S6b). This evidence suggests that citreoviridin and bortezomib did not regulate the PM calcium regulators or desensitize the ATP-induced calcium influx in 24-h treatment.

### Combination of bortezomib and citreoviridin caused caspase-independent cell death

**Figure 8:**
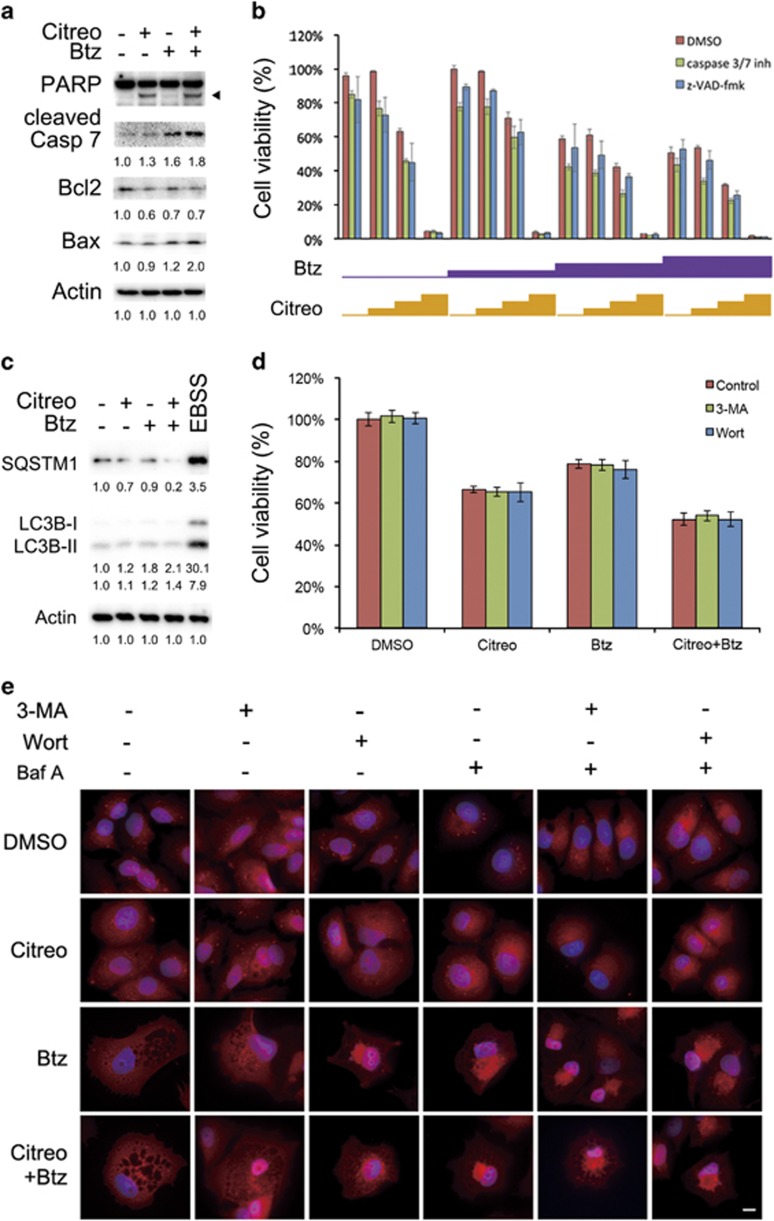
Combination of bortezomib and citreoviridin caused caspase- and autophagy- independent cell death. Cells treated with or without 0.1 *μ*M citreoviridin (Citreo) and 10 nM bortezomib (Btz) for 48 h were harvested for western blotting analysis. Expression levels of (**a**) apoptosis-related proteins and (**c**) autophagy-related proteins were normalized to actin and DMSO (−/−) levels. The black arrowhead indicates cleaved PARP. For inducing autophagy, cells were pretreated with bafilomycin A1 (Baf A) to prevent the formation of autophagolysosome and starved in EBSS for 4 h to induce autophagy. (**b**) Cells were treated with 0, 1, 10, or 100 nM Btz or with 0, 0.01, 0.1, or 1 *μ*M Citreo in the absence (i.e., DMSO only) or in the presence of 20 *μ*M pan-caspase inhibitor z-VAD-fmk or 10 *μ*M caspase 3/7 inhibitor (inh). After 48-h incubation, cell viability was measured through the MTT assay. (**d**) Cells were incubated with 5 mM 3-MA for 4 h or 1 *μ*M wortmannin (Wort) for 1 h prior to 0.1 *μ*M Citreo, 10 nM Btz, or cotreatment. The cell viability was measured by the MTT assay and normalized to the DMSO vehicle control after 48-h treatment. (**e**) MCF7 cells were seeded on to poly-L-lysine-coated slide for 24 h. Cells were pretreated with 5 mM 3-MA for 4 h, 1 *μ*M Wort for 1 h, or 100 nM Baf A for 1 h prior to 0.1 *μ*M Citreo or 10 nM Btz treatment as indicated. After a 5-day treatment, cells were subjected to probing LC3B followed by corresponding red fluorescent Alexa Fluor 555 donkey anti-rabbit IgG antibody. Cell nucleus was stained with DAPI (4′,6-diamidino-2-phenylindole; blue). White bar indicates 10 *μ*m

Next, we revealed that the combination of citreoviridin and bortezomib caused cleavage of poly ADP ribose polymerase (PARP), activation of caspase 7, decrease in B-cell lymphoma 2 (Bcl2), and increase in Bcl-2-associated X protein (Bax) (Figure 8a). However, we did not observe the typical apoptotic phenotype including phosphatidylserine (PS) exposure (Supplementary Figure S7), nuclear condensation (4′,6-diamidino-2-phenylindole (DAPI) staining in Figure 7b), and hypodiploid DNA content (Supplementary Figure S8), suggesting that the combined treatment did not cause apoptotic cell death. We then showed that cell death induced by citreoviridin and bortezomib was not prevented by co-incubation of MCF7 cells with the broad-spectrum caspase inhibitor Z-VAD-fmk or with caspase 3/7 inhibitor (Figure 8b). These results indicate that non-apoptotic cell death induced by the combination of citreoviridin and bortezomib is caspase independent.

### Combination of bortezomib and citreoviridin caused autophagy-independent cellular vacuolization and cell death

The UPR is highly connected to autophagy owing to high demand of metabolism. To examine whether the citreoviridin and bortezomib caused cell death is mediated by autophagy, we detected the protein levels of major components sequestosome 1 (SQSTM1) and microtubule-associated protein 1 light chain 3 beta (LC3B) for autophagosome formation. The levels of SQSTM1 and LC3B were markedly induced by starvation in Earle's Balanced Salt Solution (EBSS), while only slightly affected by citreoviridin and bortezomib (Figure 8c). We also demonstrated that the citreoviridin- and bortezomib-induced cell death was not rescued by 3-methyladenine (3-MA) and wortmannin (Figure 8d), indicating that PI3K activity is not required for citreoviridin- and bortezomib-inhibited cell proliferation. Furthermore, both citreoviridin and bortezomib did not induce LC3 puncta in contrast to the incremental number of LC3 puncta when cells exposed to bafilomycin A1 (Figure 8e), indicating the treatment has no effect on autophagy. Our results also demonstrated that the formation of cellular vacuolation was not affected by pretreatment of PI3K inhibitors (Figure 8e), suggesting the citreoviridin- and bortezomib-caused cytoplasmic vacuolization is independent of PI3K-mediated autophagy.

## Discussion

Ectopic ATP synthases on the surface of hepatocytes, endothelial cells, keratinocytes, and adipocytes have been revealed to regulate cholesterol uptake, angiogenesis, extracellular ATP signaling in wound healing, and preadipocyte differentiation, respectively.^31, 32, 33, 34^ The significance of ectopic ATP synthase in normal cells and cancer research has been emphasized in the past decades.^13, 14, 15, 35, 36, 37^ The expression of ectopic ATP synthase on cancer cells was first discovered in hematological cancer, the chronic myelogenous leukemia K562 cells. It acts as a ligand for developing effectors of native natural killer and interleukin 2-activated killer cells in the cytolytic pathway.^38^ In the present study, we found first evidence that oxidative phosphorylation complexes, including F_1_F_o_ ATP synthase and ETC complexes, were ectopically expressed on the PM of breast cancer cells (Figure 1). The absence of ectopic ATP synthase on the non-tumorigenic breast epithelium cells MCF10A indicates the potent role of ectopic ATP synthase in targeted therapy. The ATP synthase inhibitor citreoviridin, which targets the ATP synthase *β* subunit and inhibits ATP synthesis activity, dramatically reduced proliferation of breast cancer cells and retained the viability of non-tumorigenic MCF10A cells (Figure 2). These results suggest that ectopic ATP synthase might be crucial for the proliferation of cancer cells. Cancer cells utilize glucose rapidly and efficiently to support their unlimited growth and to develop intense switches and alterations in fundamental metabolism. Whether ectopic expression of mitochondrial proteins on the PM is involved in the dysregulation of tumor metabolism and bioenergetics in mitochondria remains unclear.

Accumulation of unfolded proteins in the ER, nutrient deprivation, calcium imbalance, oxidative stress, metabolic alterations, and environmental acidity activate UPR signaling to enable adaptation to perturbations and to relieve ER stress.^39^ Citreoviridin inhibited cell proliferation, reduced colony formation, and caused cell cycle arrest by inducing the UPR (Figures 2,3,4). This suggests the inhibition of cellular interruptions coupled to ectopic ATP synthase and therefore links the function of ectopic ATP synthase to the regulation of metabolic homeostasis in cancer cells.

As a double-edged sword, activation of UPR can lead to conflicting consequences for tumorigenesis.^40^ In response to the initial expansion of the tumor population, the UPR activates cytoprotective signaling pathways that alleviate ER stress and enhance the folding capacity to cope with excess protein production and an extreme redox environment.^40^ Angiogenesis is thus stimulated by inadequate nutrients and oxygen and is potentiated by UPR-modulated hypoxia-induced factor-1 to activate the vascular endothelial growth factor.^41, 42^ However, prolonged UPR ultimately induces cell death associated with ER stress, typically through apoptosis dependent on or independent of C/EBP homologous protein to remove overstressed cells.^43^ As the ER stress response has both protective and destructive elements, fully characterizing which branches and downstream components of the UPR are activated in tumors is essential.^40^

In parallel with the attenuation of translation and increase in chaperons, UPR signaling also activates ERAD by using the 26S proteasome for degradation of unfolded proteins. Results of our proteomic analysis showed that the identified 26S protease regulatory subunit 7 was downregulated in citreoviridin-treated cells (Supplementary Table S1). The 26S proteasome system that degrades ubiquitinated proteins in an ATP-dependent manner is composed of 20S stacked core rings and 19S regulatory particles.^44^ PSMC2, a part of the 19S regulatory complex, is responsible for degrading ubiquitinated substrates into the 20S proteasome, and its expression is essential for 19S and 26S proteasome assembly.^44, 45^ Therefore, the decrease in the 26S protease regulatory subunit 7 reduced proteasome activity and sensitized the cancer cells toward cell death.^44, 46^

As citreoviridin induced UPR, caused cell cycle arrest, and reduced cell proliferation but did not cause cell death, we then aimed to strengthen the inhibitory efficacy by testing a combined treatment. We hypothesized that irreversible ER stress could switch from signaling pathways orchestrated by cellular adaption to a dead end. To explore the role of ER stress in anticancer therapy, we combined the UPR-inducing ATP synthase inhibitor citreoviridin and the 26S proteasome inhibitor bortezomib. Both UPR signaling and cell cycle regulators were affected by the combined treatment.

To understand the regulation of the combined treatment on cell cycle progression, we examined the expression of cell cycle regulators in G_0_/G_1_, S, and G_2_/M phases. Combination with bortezomib inhibited the citreoviridin-induced compensation of cyclin D_3_/CDK6, preventing Rb phosphorylation and S-phase entry by decreasing PCNA expression (Figure 6). CDK1/cyclin B acted as the M-phase promoting factor controlling the onset of mitosis. The activity of CDK1/cyclin B is controlled by the level and localization of cyclin B and by the regulatory phosphorylation of CDK1.^47, 48^ Activation of CDK1/cyclin B is precisely regulated by its two feed-forward loops, namely, dephosphorylation of phosphorylated phosphatase Cdc25C and inhibition of Wee1 kinase.^49^ CDK1 is inactivated by Tyr15 and Thr14 phosphorylation of Wee1 and is activated by Cdc25C through dephosphorylation of Tyr15 and phosphorylation of Thr161.^50^ Here we demonstrated that induction of phosphorylation on Thr161 and Tyr15 of CDK1 and the expression of cyclin B and Cdc25C were diminished in response to the combined treatment compared with bortezomib alone. This result is similar to that of the citreoviridin treatment (Figure 6d) and accounts for the observation of accumulated populations in both G_2_/M and G_0_/G_1_ phases in response to combined treatment (Figures 6a and c).

Excessive ER stress leads to apoptotic cell death.^51^ Our results indicate that prolonged ER stress caused cell toxicity (Figure 5a), inhibited colony formation (Figure 5b), induced cleavage of PARP and caspase 7 (Figure 8a), and decreased the Bcl2/Bax ratio (Figure 8a), suggesting the triggering of apoptosis. However, typical apoptotic phenotypes, including nucleus condensation (Figure 7b), an increase in the sub-G_1_ population in cell cycle due to DNA fragmentation (Supplementary Figure S4), and exposure of PS on the cell surface (Supplementary Figure S3), did not occur. Furthermore, we observed that the combined treatment caused unconventional cytoplasmic vacuolation (Figure 7b), and caspase- and autophagy- independent cell death (Figure 8), implying that a novel type of cell death occurred. Methuosis, a novel form of non-apoptotic cell death characterized by the accumulation of vacuoles derived from macropinosomes and endosomes, was discovered and defined by Overmeyer *et al.*^52^ in 2008. Our findings in the combination treatment leading to cytoplasmic vacuolation were consistent with the morphology of glioblastoma cells overexpressing constitutive-activated RAS, which leads to methuosis. These indicate that the combination of citreoviridin and bortezomib induced methuosis-like cell death. Recently, numerous adaptive mechanisms of ER stress-induced autophagy, a process regulating homeostasis of cellular nutrients by protein and organelle turnover, have been found to contribute to the development of oncogenic properties, drug resistance in cancer therapy, and pathogenesis of chronic inflammatory diseases.^24, 53^ Furthermore, a quantitative proteomics study demonstrated that the *in vivo* anticancer activity of citreoviridin was due to the glucose metabolism switch,^54^ suggesting that citreoviridin-induced UPR might be involved in glucose metabolism.^55, 56^ Whether a relationship exists between ER stress and macropinocytosis leading to methuosis remains unknown.

In addition to ERAD, the activated UPR stimulate autophagy.^1^ Protein quality control is governed by two main processes: proteasomal degradation and autophagy.^57, 58^ Inhibition of proteasome degradation by bortezomib therefore forced the escalation of autophagy flux (Figure 8c), which is consistent with previous findings in myelomas.^59, 60, 61^ However, the use of autophagy inhibitors could not confer the citreoviridin- and bortezomib-caused cytotoxicity or cytoplasmic vacuolization (Figures 8d and e), suggesting the elevated autophagy is a minor effect along with the drug treatment. Furthermore, the perinuclear distribution of LC3B was not colocalized with the enlarged vacuoles (Figure 8e), indicating the cellular vacuolization is independent of formation of autophagosome.

In summary, our findings highlight that ectopic oxidative phosphorylation complexes are present on the PM and that ectopic ATP synthase could serve as a potential anticancer target. Our results show that the ATP synthase inhibitor citreoviridin could inhibit cancer cell proliferation by cell cycle arrest in the G_0_/G_1_ phase through activation of the UPR. An increase in ER stress caused by the 26S proteasome inhibitor bortezomib, which blocks ERAD, and citreoviridin, which targets ectopic ATP synthase, eventually resulted in non-apoptotic cell death combined with severe cytoplasmic vacuolation. With the aid of pharmaceutical proteomics and protein–protein interaction network analysis, we obtained evidence that combined therapy of inhibitors targeted collective pathways that were functionally integrated. This finding suggests that application of the combined therapy might influence diverse cellular responses and produce a coordinated outcome.

## Materials and Methods

### Cell culture

The human breast cancer cell lines MCF7 (ATCC, HTB-22), T47D (ATCC, HTB-133), and MDA-MB-231 (ATCC, HTB-26), and the mammary epithelial cell line MCF10A (ATCC, CRL-10317) were purchased from ATCC (Manassas, VA, USA). MCF7, T47D, and MDA-MB-231 cells were cultured in Dulbecco's modified Eagle's medium (DMEM, Gibco, Carlsbad, CA, USA) supplemented with 10% fetal bovine serum (Biological Industries, Beit Haemek, Israel). MCF10A cells were grown in MEBM (Lonza, Baltimore, MD, USA) supplemented with 5% fetal bovine serum (Gibco), 5 mg/l insulin (Sigma-Aldrich, St Louis, MO, USA), 5 mg/l hydrocortisone (Sigma-Aldrich), and 10 *μ*g/l human epidermal growth factor (Sigma-Aldrich). All cell lines were maintained at 37 °C in humidified air with 5% CO_2_ and routinely passaged at 85–90% confluence. All cells were free of mycoplasma, as determined through a PCR-based mycoplasma detection method (MBI Fermentas, St Leon-Rot, Germany).

### Drug treatment

The ATP synthase inhibitor citreoviridin (Enzo Life Sciences, Farmingdale, NY, USA) and the proteasome inhibitor bortezomib (Biovision, Mountain View, CA, USA) were solubilized in dimethyl sulfoxide (DMSO) to prepare a 20 mM stock solution and which was then freshly diluted in medium to specified concentrations. Control samples were treated with the same volume of DMSO (Sigma-Aldrich). The final concentration of DMSO in the medium was 0.1%. All procedures, including drug preparation and treatment, were carried out in the dark. Wortmannin (Cayman Chemical Co., Ann Arbor, MI, USA) and bafilomycin A1 (Cayman Chemical Co.) were dissolved in DMSO to 1 mM stock. Five molar stock of 3-MA (Cayman Chemical Co.) was freshly prepared before use in 55 °C sterile deionized water. For inhibiting autophagy, cells were pretreated with 5 mM 3-MA for 4 h or 1 *μ*M wortmannin for 1 h to block the PI3K activity or 100 nM bafilomycin A1 for 1 h to prevent proceeding of late autophagy. For inducing autophagy, cells pretreated with 100 nM bafilomycin A1 for 1 h were incubated in EBSS (Invitrogen, San Diego, CA, USA) for 4 h subsequently.

### Immunofluorescence staining

Cells (5 × 10^4^) were seeded on a glass coverslip that was coated with 0.1% poly-L-lysine (Sigma-Aldrich) and then allowed to adhere overnight. The cells were fixed with 4% paraformaldehyde (Sigma-Aldrich) for 10 min, washed three times with PBS, and incubated with 5% BSA in PBS for 30 min to block the non-specific binding of antibodies. For detecting the mitochondrial localized proteins, cells were permeablized with 0.25% Triton X-100 in PBS for 10 min at room temperature and washed three times with PBS before the blocking step. Cells were subsequently incubated with the monoclonal antibody-probing NDUFB4 (Abcam Inc., Cambridge, MA, USA), SDHA (Abcam Inc.), UQCRC2 (Abcam Inc.), COX5A (Abcam Inc.), ATP synthase *β* (Abcam Inc.), ATP synthase complex (Abcam Inc.), and calnexin (Cell Signaling Technology, Danvers, MA, USA) at 4 °C overnight or/and LC3B (Cell Signaling Technology) at 37 °C for 3 h. After the cells were washed three times with PBS, they were incubated with secondary anti-mouse IgG-Alexa 488 (Invitrogen) or anti-rabbit IgG-Alexa 555 (Invitrogen) for 30 min in the dark. The cells were then washed three times with PBS. The immunostained cells were incubated with DAPI (Sigma-Aldrich) for 10 min in the dark. Subsequently, they were mounted with ProLong Gold antifade reagent (Invitrogen) and examined under a fluorescence microscope using a Leica HCX FL PLAN 100 × 1.25 OIL objective (Leica Lasertechnik, Heidelberg, Germany).

### Real-time measurement of cell growth and 3-(4,5-cimethylthiazol-2-yl)-2,5-diphenyl tetrazolium bromide (MTT) assay

The xCELLigence real-time cell analyzer (RTCA) System from Roche Applied Science (Roche Applied Sciences, Indianapolis, IN, USA) was used in label-free, continuous monitoring of cell proliferation after citreoviridin treatment, according to the manufacturer's instructions. Cells (1 × 10^4^ per well) were seeded on a RTCA plate, cultured in a CO_2_ incubator at 37 °C and 5% CO_2_, and continuously monitored with the xCELLigence DP system. Approximately 24 h after cell seeding, cells in the exponential growth phase were treated with various concentrations of citreoviridin. The cell response to the citreoviridin treatment was monitored continuously for up to 48 h. Mean and S.D. values for each concentration and combination were imported into the Calcusyn software (Biosoft, Ferguson, MO, USA) to perform the combination index simulations and to analyze the synergy at different dosage levels, where combination index <1 indicates synergy and >1 indicates antagonism. For the MTT assay, 1 × 10^4^ cells were plated in 96-well plates and allowed to adhere overnight. The medium was then discarded, and the cells were pretreated with 20 mM Z-VAD-fmk (a pan-caspase inhibitor) or with freshly prepared 10 mM caspase 3/7 inhibitor for 1 h. Treatment with citreoviridin and bortezomib at the indicated combinations and concentrations was performed for a further 48 h. To investigate the involvement of autophagy, cells were pretreated with 5 mM 3-MA for 4 h or 1 *μ*M wortmannin for 1 h prior to 48-h incubation of 0.1 *μ*M citreoviridin, 10 nM bortezomib, or cotreatment. Cell survival was measured through the MTT assay, as described previously.^13^ The percentage of viability refers to the MTT value for 0.1% DMSO-treated cells.

### Cell cycle analysis

Cells were trypsinized and fixed overnight with 70% ethanol at −20 °C. They were incubated with 50 *μ*g/ml RNase A (Santa Cruz Biotechnology, Santa Cruz, CA, USA) in PBS for 30 min at room temperature. Propidium iodide (10 *μ*g/ml, Santa Cruz Biotechnology) was added, and the DNA content of the cells was analyzed on a FACSCanto instrument (Becton Dickinson, San Jose, CA, USA). The percentages of cells in different phases of the cell cycle were measured by using ModFit (Verity Software House, Topsham, ME, USA).

### Protein extraction

The cells were harvested and dissolved in lysis buffer containing 7 M urea (Boehringer, Mannheim, Germany), 2 M thiourea, 4% 3-[(3-cholamidopropyl)dimethylammonio]-1-propanesulfonate (CHAPS) (JT Baker, Phillipsburg, NJ, USA), and 0.002% bromophenol blue (Amersco, Solon, OH, USA). The mixture was sonicated for 2 min on ice in 0.6 on/0.4 off cycle per seconds with an ultrasonic probe (Labsonic M; Sartorius, Tagelswangen, Switzerland). The lysates were centrifuged at 12 000 × *g* for 30 min at 4 °C. Supernatants were collected, and protein concentrations were determined by using a protein assay kit (Bio-Rad, Hercules, CA, USA). The protein samples were stored at −80 °C until use.

### Western blotting

Proteins were separated on SDS polyacrylamide gels and then transferred to polyvinyldifluoride (PVDF) membranes (Millipore, Billerica, MA, USA). PVDF membranes were blocked with 5% milk in phosphate-buffered saline containing 0.1% Tween-20 and then incubated with antibody-probing-specific antigens. Antibody-probing PERK, IRE1*α*, BiP, Ero1-L*α*, PDI, phospho-eIF2*α*, eIF2*α*, cyclin D_1_, cyclin D_3_, CDK6, cleaved caspase 7, and LC3B were purchased from Cell Signaling Technology. Antibodies detecting cyclin B_1_, Cdc25C, p27, CDK4, PCNA, SQSTM1, and *β*-actin were obtained from GeneTex (Irvine, CA, USA). Phospho-Rb and Rb antibodies were obtained from Epitomics (Burlingame, CA, USA), and p53, p21, phospho-CDK1 (T161), and CDK1 antibodies were acquired from Santa Cruz Biotechnology. After incubation with secondary antibodies, immunoblots were reacted with an ECL detection kit (Millipore), detected by a FluorChem M Multifluor System (ProteinSimple, San Jose, CA), and then quantified by using the Kodak 1D Image Analysis software version 3.6 (Kodak, New Haven, CT, USA).

### siRNAs and transfection

MCF7 cells (1 × 10^5^) were incubated in six-well culture plates for 24 h before transfection. The confluency of the cells at the time of transfection was 30–50%. Cells were transfected with 80 pmol of the control or with PERK siRNA (Santa Cruz Biotechnology) with 7.5 *μ*l Lipofectamine 2000 (Invitrogen) in serum-free DMEM according to the manufacturer's instructions. After transfection for 4 h at 37 °C, the medium was replaced with DMEM containing 10% FBS. After 36 h of transfection, cells were treated with 0.1% DMSO or with 0.1 *μ*M citreoviridin. Cells were collected after 24 h of treatment and then stored at −80 °C until protein analysis.

### Clonogenic assay and soft agar assay

For the clonogenic assay, cells were plated at a density of 2000 cells/well in six-well culture plates. After 24 h, cells were treated with either 0.1% DMSO or 0.1 *μ*M citreoviridin, followed by an additional 10-day incubation to allow colony formation. Colonies were fixed and stained with 0.05% crystal violet (Sigma-Aldrich), washed to remove excess dye, and then imaged by a scanner (Scanjet 4500c, Hewlett-Packard, Palo Alto, CA, USA). Quantitative changes in clonogenicity were determined by counting the number of colonies. For the soft agar assay, a 2-ml mixture of complete DMEM and 0.7% low-melting-temperature agarose (Seaplaque: Lonza, Rockland, ME, USA) was added to a six-well tissue culture dish and then allowed to solidify (base agar). Next, a 2-ml mixture of complete DMEM and 0.35% agar containing 0.1 *μ*M citreoviridin and 5000 cells was added to the top of the base layer; this layer was allowed to solidify (top agar). The cells were cultured for 20 days at 37 °C in an incubator with a humidified atmosphere containing 5% CO_2_. At the end of the incubation, cell colony formation was assessed by a colorimetric assay using crystal violet. Colonies were photographed under × 40 magnification.

### Statistical analyses

All data are presented as the mean±S.D. of at least three independent experiments. Statistical analyses were performed by using unpaired two-tailed *t*-tests to compare the two groups. *P*-values that were <0.05 were considered significant.

## Abbreviations

ATF6: activating transcription factor 6
ATP: adenosine triphosphate
Bax: Bcl-2-associated X protein
Bcl2: B-cell lymphoma 2
CDK: cyclin-dependent kinase
CKI: cyclin-dependent kinase inhibitor
eIF2*α*: eukaryotic translation initiation factor 2*α*
ER: endoplasmic reticulum
ERAD: ER-associated degradation
ETC: electron transport chain
Ero1-L*α*: ER-residing protein endoplasmic oxidoreductin-1-like *α*
IRE1: inositol-requiring protein-1
LC3B: microtubule-associated protein 1 light chain 3 beta
MMP: mitochondrial membrane potential
PARP: poly ADP ribose polymerase
PCNA: proliferating cell nuclear antigen
PERK: protein kinase RNA-like endoplasmic reticulum kinase
PM: plasma membrane
PS: phosphatidylserine
SQSTM1: sequestosome 1
siRNA: small interfering RNA
UPR: unfolded protein response
