# Supplementary material for: Combination therapy targeting ectopic ATP synthase and 26S proteasome induces ER stress in breast cancer cells
Source: Cell Death Dis. 2014 Nov 27;5(11):e1540–. doi: 10.1038/cddis.2014.504 (PMC4260757; doi:10.1038/cddis.2014.504)
Supplement: Supplementary Information [file cddis2014504x1.doc]

**Supplementary Information**

**Combination Targeting of Ectopic ATP Synthase and 26S Proteasome Enhances ER stress against breast cancer cells**

Hsin-Yi Chang^1^, Tsui-Chin Huang^2^, Nai-Ning Chen^3^, Hsuan-Cheng Huang^4,*^, Hsueh-Fen Juan^1,3,5,*^

^1^Department of Life Science, National Taiwan University

^2^Ph.D. Program for Cancer Biology and Drug Discovery, College of Medical Science and Technology, Taipei Medical University

^3^Institute of Molecular and Cellular Biology, National Taiwan University

^4^Institute of Biomedical Informatics, Center for Systems and Synthetic Biology, National Yang Ming University

^5^Graduate Institute of Biomedical Electronics and Bioinformatics, National Taiwan University

^*^Correspondence: H-C Huang, Institute of Biomedical Informatics and Center for Systems and Synthetic Biology, National Yang-Ming University, No.155, Sec.2, Linong Street, Taipei 112, Taiwan. Tel: +886-2-28267357; Email: [hsuangcheng@ym.edu.tw](mailto:hsuangcheng@ym.edu.tw); H-F Juan, Department of Life Science, Institute of Molecular and Cellular Biology, Graduate, Institute of Biomedical Electronics and Bioinformatics, National Taiwan University, No. 1, Sec. 4, Roosevelt Rd., Taipei 116, Taiwan. Tel: +886-2-33664536; Fax: +886-2-23673374; E-mail: [yukijuan@ntu.edu.tw](mailto:yukijuan@ntu.edu.tw)

**Supplementary information includes:**

**1. Supplementary Materials and Methods**

**2. Legends to Supplementary Figures**

**Supplementary Materials and Methods**

*Biotin labeling and purification of plasma membrane proteins*

Biotionylation of plasma membrane (PM) proteins were performed when cells growth reached to 70%-90% confluency in T75 flasks. Four T75 flasks of cells were washed with iced PBS three times, and incubated with freshly prepared 0.25 mg/ml EZ-Link Sulfo-NHS-SS-Biotin (Pierce, Rockford, IL, USA) in PBS for 1 h at 4°C. Exceed free biotin was quenched by addition of Tris-HCl (pH 7.5) to a final concentration of 50 mM. The cells were scraped and washed three times with Tris buffered saline. Cells were lyzed with 0.5 ml of lysis buffer (50 mM sodium phosphate buffer (pH 8.0), 150 mM NaCl, 1% Nonidet P-40, 0.5% Triton X-100) with protease inhibitor and incubated at 4°C for 30 min after disruption with 5 strokes using a probe sonicator (Sartorius, Labsonic M, Germany). Unbroken cells and nuclei were pelleted from the cell homogenate by centrifugation at 12000 x g for 10 min at 4°C. The clarified supernatant was incubated with 500 μl of NeutrAvidin (Pierce) for 1 h at 4°C. The beads were washed three times with cell lysis buffer to remove non-specific binding and the biotinylated proteins were eluted with 500 μl SDS sample buffer (62.5 mM Tris, pH 6.8, 1% SDS, 10% glycerol, 50 mM DTT).

*Two-dimensional electrophoresis (2DE) analysis*

2DE was performed by using Ettan IPGphor (Amersham Biosciences, Uppsala, Sweden). Total proteins at 500 μg were mixed with 315 μl rehydration buffer containing 7 M urea (Boehringer, Mannheim, Germany), 2 M thiourea (JT Baker, Phillipsburg, NJ, USA), 4% CHAPS (J. T. Baker), 65 mM DTE (J. T. Baker), 0.5% pH 3–10 NL IPG Buffer (Bio-Rad, Hercules, CA, USA), and 0.002% bromophenol blue (Amersco). The mixtures were incubated for 4 hours at 4°C, centrifuged at 12,500 x g, and loaded onto an 18 cm pH 4–7 or 3-10 NL gradient Immobiline DryStrip (Bio-Rad). The strips were rehydration at 50 V for at least 12 hours and IEF was carried out using the following conditions: (1) 100 V for 2 hours; (2) 250 V for 1 hour; (3)500 V for 1 hour; (4)1000 V for 1 hour; (5) 4000 V for 1 hour; and (6) 8000 V for 65,000 Vhour. After reduction with 65 mM DTE and alkylation with 55mM iodoacetamide, the second-dimensional separation was performed on a gradient 10–15% polyacrylamide gel. The protein gels were fixed in 10% methanol / 7% acetic acid, and stained using the SYPRO Ruby (Invitrogen Corporation, Carlsbad, CA). After destained by fix buffer, gels were then scanned using a Typhoon 9400^TM^ Fluorescence Imager (Amersham Biosciences) and analyzed using the Image Master 2D elite software package (Amersham Biosciences) with high image quality TIF format.

*In gel digestion and peptide extraction*

The protein spots of interests were excised from the gel. The gel pieces were destained twice with 1:1 (v/v) solution containing 50 mM ammonium bicarbonate and acetonitrile (ACN). The destained gel pieces were dehydrated with 100% ACN and were then air-dried. Proteins were digested for 16 hours at 37°C with sequence-grade trypsin (Promega Corporation, WI). The resulting peptides were extracted from the gel with 1% trifluoroacetic acid (TFA) in 50% ACN twice. The combined extracts were evaporated to dryness.

*Protein identification*

The protein fragments were dissolved in 0.1% TFA and directly spotted onto the sample plate of a MALDI-TOF mass spectrometer. MALDI-TOF MS analysis was performed on a dedicated Q-Tof Ultima MALDI instrument (Micromass, Manchester, U.K.) with fully automated data directed acquisition using predefined probe motion pattern and peak intensity threshold. All individual MS data thus generated from a particular sample well were then output as a single MASCOT-searchable peak list file. Within each sample well, parent ions that met the predefined criteria (any peak within the m/z 800–4000 range with intensity above 10 count. Subsequently, protein identification was determined by searching in the Swiss-Prot version 51.7 database using the MASCOT (http:// www.matrixscience.com) search engine as previously described^38^. All the searching parameters were set as follows: peptide mass tolerance was 100 ppm; modifications were carbamidomethylation (C) and oxidation of methionine.

*Apoptosis analysis*

Cells were treated with DMSO, 0.1 μM citreoviridin (Citreo), 10 nM bortezomib (Btz), or combination of Citreo and Btz for 48 hours and 5 × 10^5^ cells were harvested for apoptosis analysis. Apoptotic and necrotic cell death was recongnized by staining with fluorescein isothiocyanate (FITC)-conjugated annexin V (AV) and propidium iodide (PI), respectively. Double staining was performed according to the manufacturer's instructions (BD Biosciences, San Jose, CA, USA). The intensities of green and red fluorescence were measured with a FACS Canto II flow cytometer (BD Biosciences). The proportion of viable (AV^−^/PI^−^), apoptotic (AV^+^/PI^−^), necrotic (AV^+^/PI^+^) cells, and the dead (AV^+^/PI^+^) cells was calculated using the FACSDiva software (BD Biosciences).

*Measurement of mitochondrial membrane potential*

MCF7 cells were treated with 0.1 μM citreoviridin or 10 nM bortezomib for 48 hours. 10^6^ cells were harvested and stained with 2 μM JC-1 at 37^o^C for 30 minutes in the dark. For positive control, cells were then treated with 50 nM FCCP for 10 minutes at 37^o^C to depolarize the mitochondrial membrane potential. The fluorescent intensity of JC-1 green monomers and red aggregates was detected by a flow cytometer (FACSCanto II; BD Biosciences).

*Detection of calcium flux*

MCF7 cells were washed with HBSS (containing 20 mM HEPES pH7.2, 2 mM CaCl_2_, 1 mM MgSO_4_, 1 mM probenecid) and loaded with the cell-permeant fluorescent calcium indicator, Fluo-4 AM (4 μM) for 30 minutes at room temperature. After washed off the extracellular dye, cells were incubated at 37^o^C for 60 minutes for de-esterification. Changes in fluorescence were detected using a Flexstation 3 microplate reader (Molecular Devices) for 315 seconds in 2 seconds of interval. Fluorescence was excited at 485/20 nm and emission was measured at 525/20 nm. Intracellular Ca^2+^ transients were calibrated to the minimal calcium fluorescence baseline produced by EGTA (10 mM, 0% response).

**Supplementary Table and Figures**

Table S1. Differentially expressed proteins identified by mass spectrometry are listed in table.

| **Spot** | **Protein name** | **Accession number** | **Gene name** | **Score** | **Coverage** | **Match peptide** | **MW (Da)** | **pI** | **Ratio (T/C)** | **E value** |
| --- | --- | --- | --- | --- | --- | --- | --- | --- | --- | --- |
| **1** | Heat shock 70 kDa protein 1A/1B | P08107 | HSPA1A | 91 | 42% | 20 | 70009 | 5.48 | 0.54 | 1.50E-05 |
| **2** | Tubulin beta chain | P07437 | TUBB | 110 | 38% | 20 | 49639 | 4.78 | 1.15 | 2.00E-07 |
| **3** | Endoplasmin | P14625 | HSP90B1 | 76 | 23% | 24 | 92411 | 4.76 | 0.58 | 0.00057 |
| **4** | Serine/threonine-protein phosphatase 2A 65 kDa regulatory subunit A alpha isoform | P30153 | PPP2R1A | 75 | 31% | 17 | 65627 | 5 | 0.81 | 0.00069 |
| **5** | 26S protease regulatory subunit 7 | P35998 | PSMC2 | 81 | 45% | 17 | 48603 | 5.71 | 0.76 | 0.00017 |
| **6** | Rab GDP dissociation inhibitor beta | P50395 | GDI2 | 63 | 40% | 15 | 50631 | 6.11 | 0.2 | 0.0097 |
| **7** | Heat shock protein beta-1 | P04792 | HSPB1 | 87 | 59% | 12 | 22768 | 5.98 | 0.87 | 4.10E-05 |
| **8** | 78 kDa glucose-regulated protein | P11021 | HSPA5 | 108 | 40% | 22 | 72288 | 5.07 | 2.14 | 8.30E-06 |
| **9** | 78 kDa glucose-regulated protein | P11021 | HSPA5 | 197 | 49% | 31 | 72288 | 5.07 | 1.73 | 4.00E-16 |
| **10** | Gelsolin | P06396 | GSN | 70 | 26% | 19 | 85644 | 5.9 | 1.77 | 0.002 |
| **11** | T-complex protein 1 subunit beta | P78371 | CCT2 | 98 | 47% | 19 | 57452 | 6.01 | 0.54 | 3.40E-06 |
| **12** | Alpha-enolase | P06733 | ENO1 | 149 | 57% | 24 | 47139 | 7.01 | 0.14 | 2.60E-11 |
| **13** | 14-3-3 protein epsilon | P62258 | YWHAE | 70 | 51% | 14 | 29155 | 4.63 | 3.96 | 0.002 |
| **14** | 14-3-3 protein zeta/delta | P63104 | YWHAZ | 72 | 49% | 13 | 27728 | 4.73 | 1.65 | 0.0013 |
| **15** | Proteasome activator complex subunit 1 | Q06323 | PSME1 | 72 | 53% | 14 | 28705 | 5.78 | 2.61 | 0.0014 |


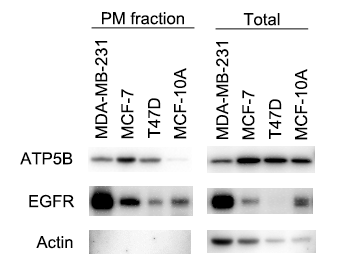


**Figure S1. Expression of ATP synthase β on plasma membrane of breast cancer cells.** Biotinylation of plasma membrane protein were performed to purify plasma membrane localized proteins. 20 μl elutes (PM) and 2 μl total cell lysates (Total) were analyzed by immunoblotting. Expression of EGFR was used as the control for plasma membrane localized protein, and that of Actin was applied to estimate the purification specificity.


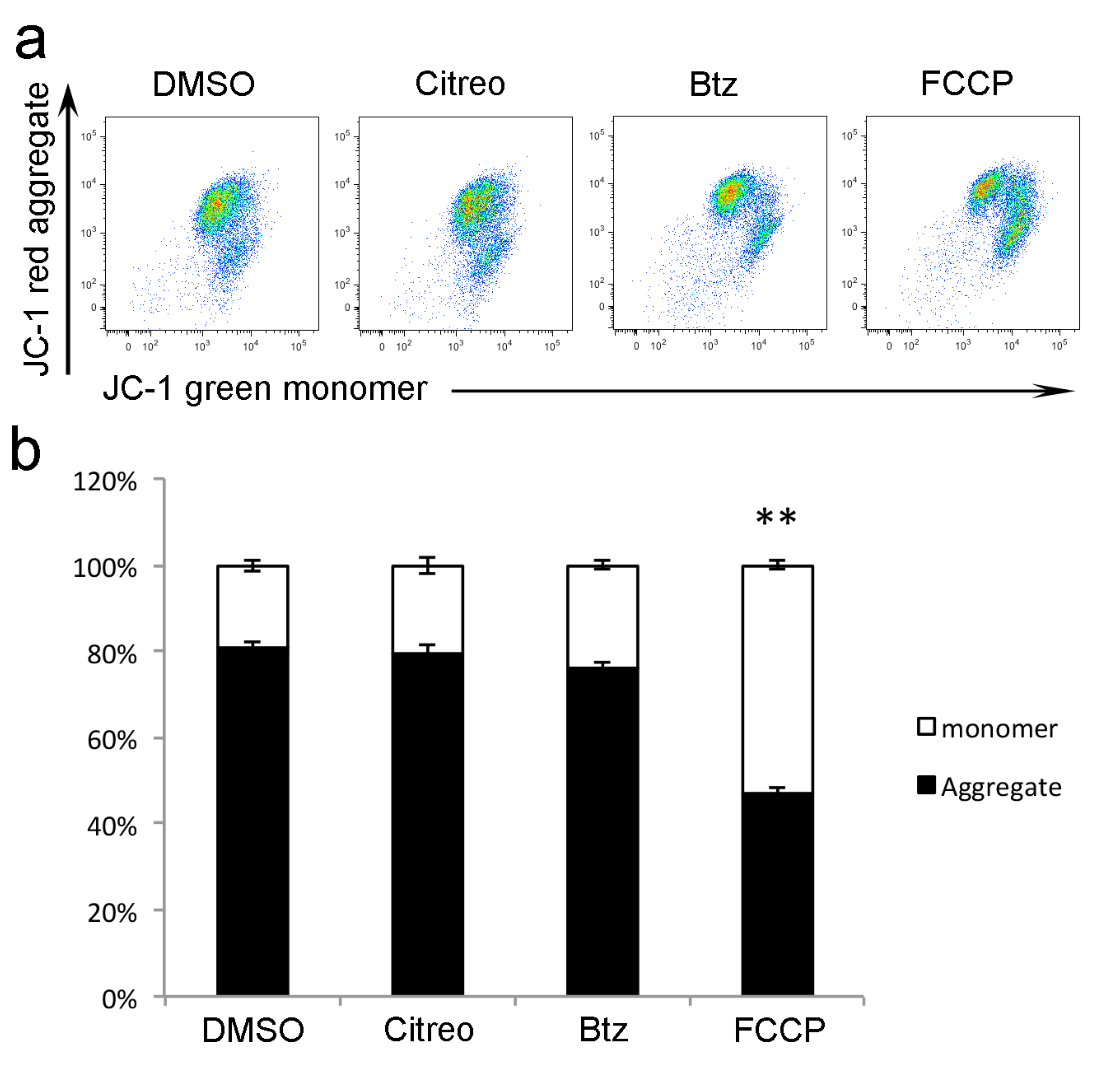


**Figure S2. Detection of mitochondrial membrane potential.** MCF7 cells were incubated with 0.1 μM citreoviridin (Citreo), 10 nM bortezomib (Btz) or cotreatment for 48 hours. 10^6^ cells were counted and stained with JC-1. FCCP treated cells were used as the positive control for mitochondrial membrane potential depletion. (a) The fluorescent intensity of JC-1 green monomers and red aggregates was detected by a flow cytometer (FACSCanto II; BD Biosciences). (b) Statistics analysis was performed from three independent experiments. ** indicates *P* < 0.001 compared to DMSO control.


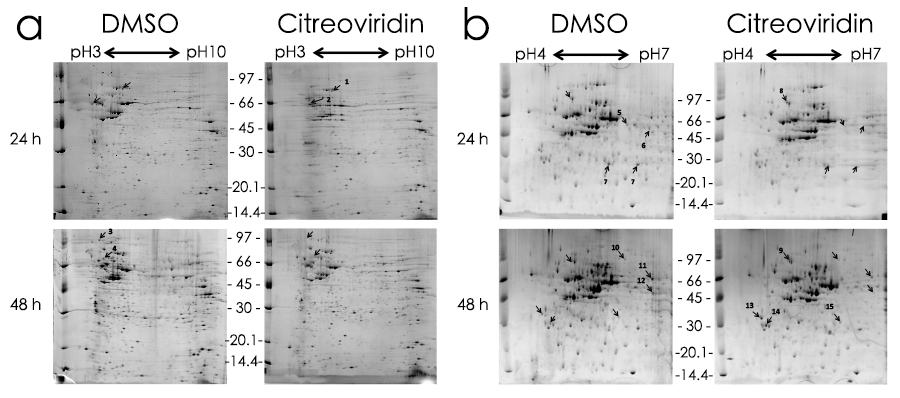


**Figure S3. Proteomics analysis of citreoviridin treated MCF7 cells.** 500 μg protein extracted from 24-hour (upper) and 48-hour (lower) DMSO or 0.1 μM citreoviridin treated cells were separated in (a) pH 3 to 10 and (b) pH 4 to 7 for IEF and subsequently in 10-15% gradient SDS-PAGE. Differentially expressed proteins indicated by arrows were further identified by MALDI-TOF and the detailed information is listed in Table S1.

**
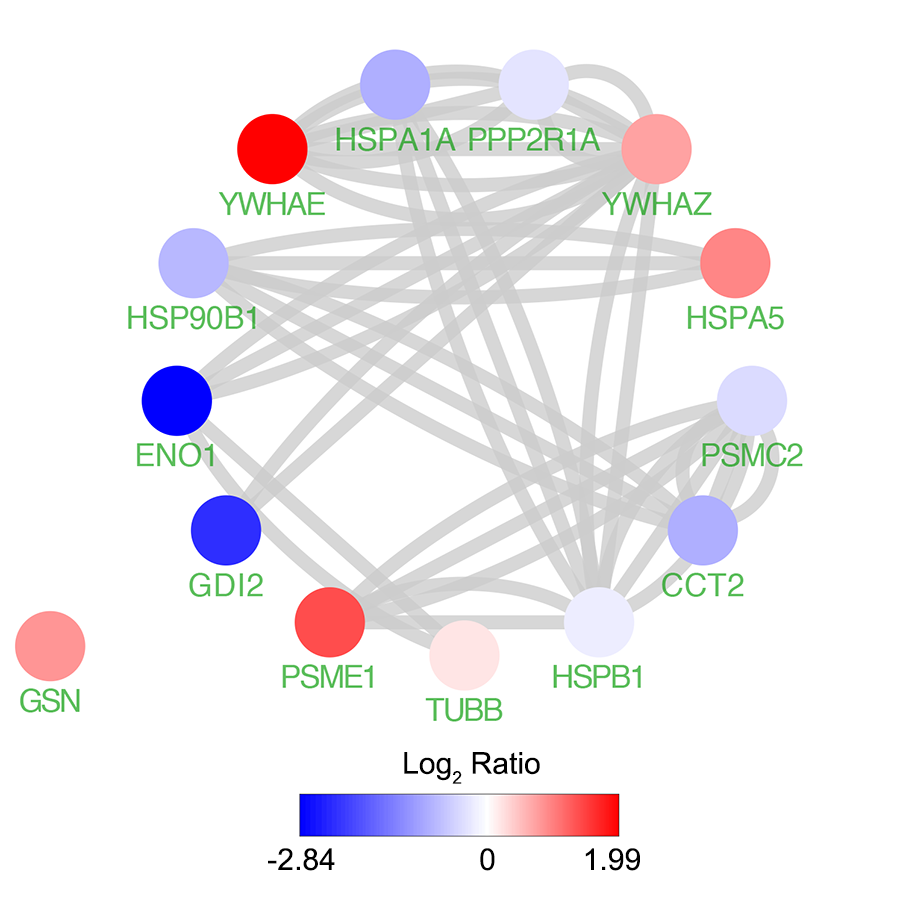
**

**Figure S4. Protein-protein interaction network (PPIN) of differentially expressed proteins.** Proteins identified by proteomics study were subjected to STRING 9.0 web tool for PPIN analysis. Proteins labeled in gene symbol (green) are gradually colored according to their expression fold change (log_2_ ratio) from 1.99 (red) to -2.84 (blue). Gray lines represent interacting evidence from literatures or high throughput experimental results.


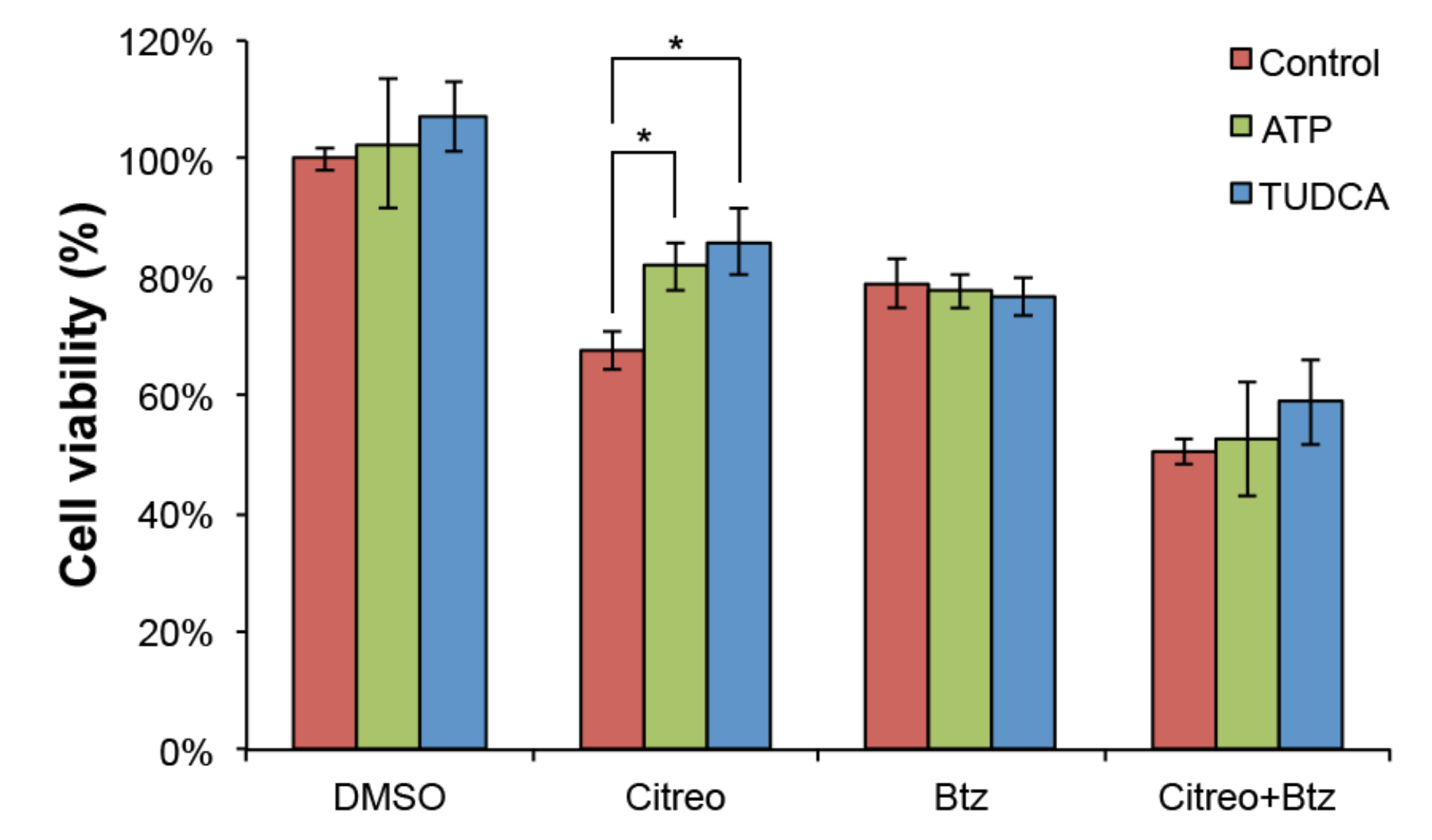


**Figure S5. Attempted rescue of citreoviridin-induced growth inhibition.** 10 μM ATP was administered 5 minutes after or 10 μM chemical chaperone tauroursodeoxycholic acid (TUDCA) was applied for 1 hour prior 0.1 μM citreoviridin (Citreo) or 10 nM bortezomib (Btz) treatment. The cell viability was measured by the MTT assay and normalized to the DMSO vehicle control treatment after 48-hour treatment. Asterisks indicate significant differences from the control (red bar) group (*P* < 0.05).


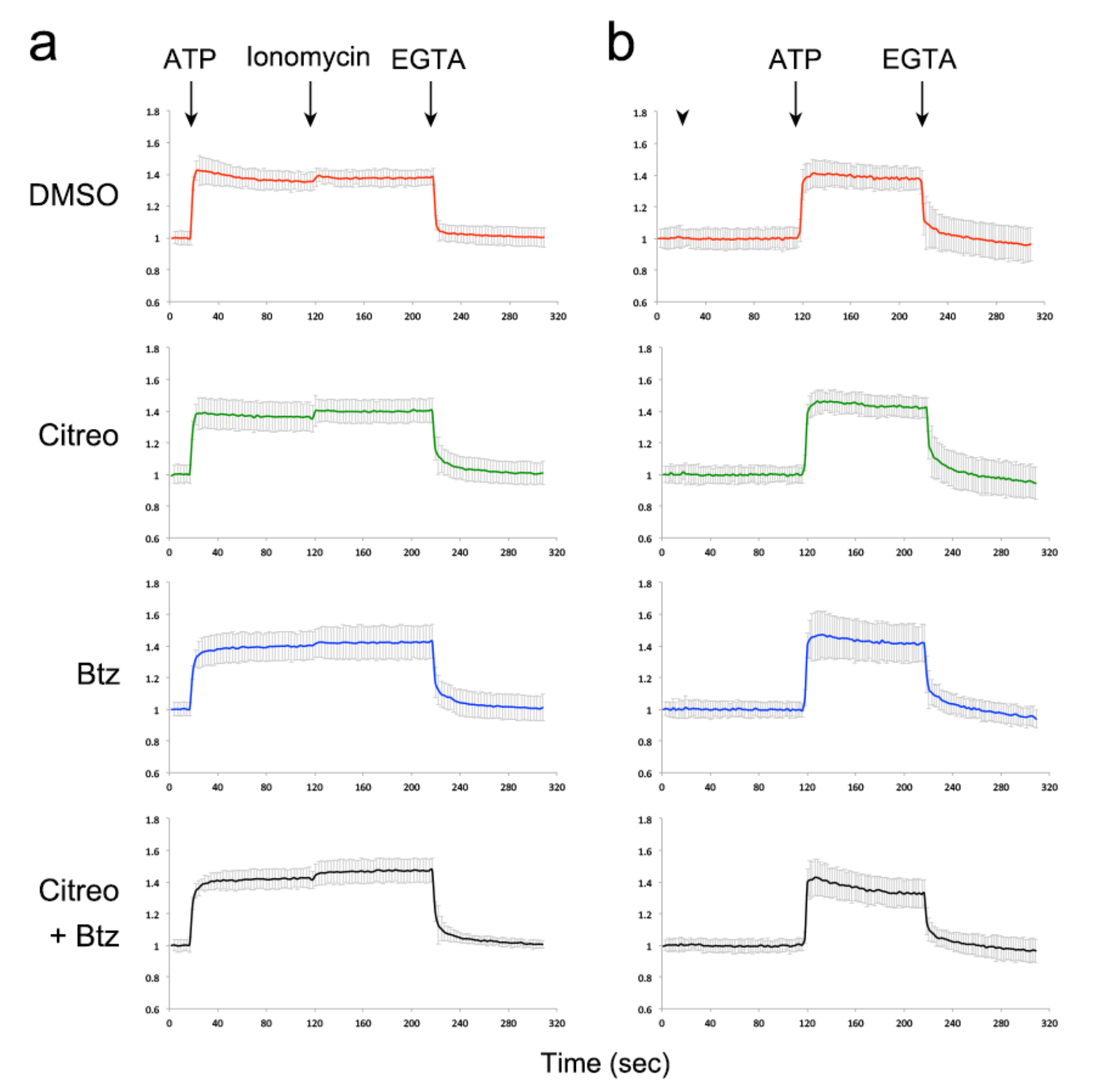


**Figure S6. Calcium influxes in MCF7 cells.** Cells stimulated by ATP (10 mM) were tested after (a) 24-hour pretreatment or (b) instant treatment of the cells with 0.1% DMSO, 0.1 μM citreoviridin (Citreo), 10 nM bortezomib (Btz), and the combined treatment. Arrows indicate the time of compounds application. Arrowhead indicates the time of drug addition. Note that no changes in baseline fluorescence were observed after application of citreoviridin and bortezomib. Intracellular Ca^2+^ transients were calibrated to the minimal calcium fluorescence baseline produced by EGTA (y-axis, 10 mM, 0% response). Each point represents pooled data from six experiments. The vertical gray bars represent standard deviation. The results were repeated in three independent experiments.

**
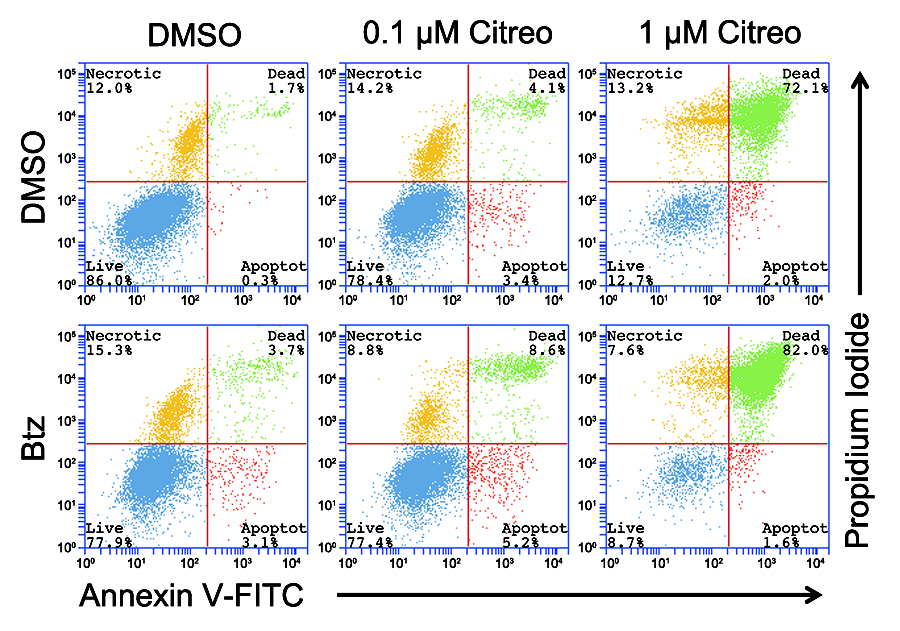
**

**Figure S7. Apoptosis analysis of citreoviridin and bortezomib caused cell death.** MCF7 cells treated with 0, 0.1, or 1 μM citreoviridin (Citreo) in presence or absence of 10 nM bortezomib (Btz) were harvested and stained with FITC-annexin-V (AV) and PI for 15 minutes. The signals of AV (x-axis) and PI (y-axis) were analyzed by a flow cytometer. The proportion of dead (quadrant 1), necrotic (quadrant 2), live (quadrant 3), and apoptotic (quadrant 4) cells were gated according to unstained control.

**
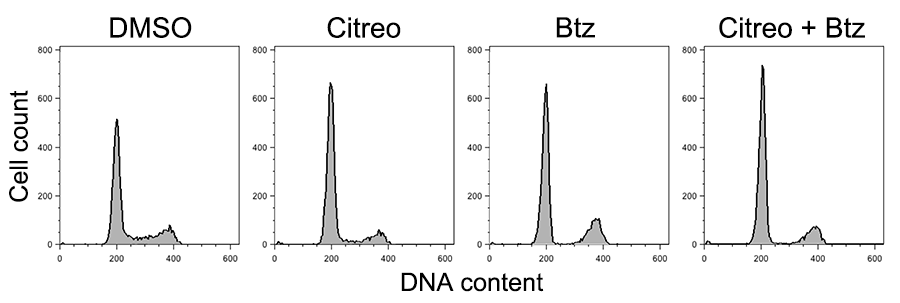
**

**Figure S8. Cell cycle analysis of citreoviridin and bortezomib treated MCF7 cells.** Cells were incubated with 0.1 μM citreoviridin (Citreo) or 10 nM bortezomib (Btz) for 48 hours. After fixed in cold ethanol, incubated with RNase A, and stained with PI, linear signal of DNA content (x-axis) was analyzed with a flow cytometer.
